# Effects of 34 Weeks of Military Service on Body Composition and Physical Fitness in Military Cadets of Angola

**DOI:** 10.3390/jfmk9030111

**Published:** 2024-06-26

**Authors:** Manuel Coge, Henrique Pereira Neiva, Ana Pereira, Luís Faíl, Bruno Ribeiro, Dulce Esteves

**Affiliations:** 1 Department of Sport Sciences, University of Beira Interior, Convento de Santo António, 6201-001 Covilhã, Portugal; mcoge@hotmail.com (M.C.); luisfail_93@hotmail.com (L.F.); ribeiro.aikido@gmail.com (B.R.); desteves@ubi.pt (D.E.); 2 Research Center in Sports Sciences, Health Sciences and Human Development (CIDESD), Convento de Santo António, 6201-001 Covilhã, Portugal; 3 Instituto Politécnico de Setúbal, Escola Superior de Educação, Departamento de Ciências e Tecnologias, Campus do IPS, Estefanilha, 2914-504 Setúbal, Portugal; ana.fatima.pereira@ese.ips.pt; 4 SPRINT Sport Physical Activity and Health Research & Innovation Center, Centro de Investigação e Inovação em Desporto Atividade Física e Saúde, 2001-904 Santarém, Portugal

**Keywords:** soldiers, anthropometrics, cardiorespiratory fitness, strength, training

## Abstract

Military personnel need physical fitness to effectively carry out operational military activities within their specific field of operation. This research investigates the effects of a 34-week training program on Angolan cadets’ body composition, muscle strength, and cardiorespiratory fitness. Seventy-four volunteer recruits, aged 18 to 26 years, were monitored during their eight-month military service, following an exercise program protocol comprising 12 weeks of strength training followed by 24 weeks of endurance training. Anthropometric variables, including body mass, body mass index, and fat mass, were assessed, along with cardiorespiratory fitness (VO_2_max), sprint performance, countermovement jump (CMJ), medicine ball throw, push-ups, and curl-ups. The physical training protocol encompassed running sessions, strength exercises, agility drills, and flexibility exercises. The initial assessment revealed gender differences in various parameters such as body mass, body fat percentage, VO_2_max, sprinting, countermovement jump (CMJ), medicine ball throw, and push-ups. Following the training program, changes were observed in all variables (effect size between 0.48 and 2.33, *p* < 0.01) for the participants. Significant interactions (sex × time) were found in body mass (F = 5.18, *p* = 0.03, ηp^2^ = 0.06), body fat percentage (F = 5.31, *p* < 0.01, ηp^2^ = 0.14), and medicine ball throw (F = 10.84, *p* < 0.01, ηp^2^ = 0.13). Specifically, males exhibited a greater reduction in body mass (females: 2.70%, males: 3.47%, *p* < 0.05) and a substantial improvement in ball throwing performance (females: 7.74%, males: 11.47%, *p* < 0.01), while females experienced a greater reduction in fat mass (females: 5.34%, males: 3.15%, *p* < 0.01). The physical training regimen effectively influenced body composition, particularly in enhancing strength performance among males. The integration of exercise programs with military service led to a notable reduction in fat tissue and an increase in lean tissue. Hence, the development of tailored training protocols is imperative to enhance the physical capacity and overall health of military recruits, considering sex-specific characteristics and physical attributes.

## 1. Introduction

The volatile military-political landscape worldwide, coupled with concurrent international armed conflicts across various regions and the ongoing development of new methods and weaponry, underscores the imperative to bolster defense capabilities for safeguarding sovereignty, territorial integrity, and inviolability. This necessitates a heightened emphasis on the professional training of military personnel [1]. Despite the advancements in weaponry, it remains evident that the effectiveness of any armament ultimately relies on the skill and expertise of those who wield it [2]. In modern warfare, service members are assigned a wide range of combat missions, often in challenging environmental conditions and amidst the hardships of combat. This includes prolonged shifts in confined spaces, demanding physical exertion, emotional stress, and the execution of multifaceted tasks under challenging circumstances. Such scenarios require comprehensive physical and mental preparedness, encompassing endurance, strength, agility, and adaptability to effectively operate in any situation, terrain, or time of day [3,4]. Physical performance is directly related to operational capability as it enables service members to endure long hours, maintain high levels of alertness and readiness, and execute complex tasks efficiently. Improved physical fitness enhances resilience against the physical and mental stresses of combat, ensuring that service members can perform their duties effectively and sustain peak performance under extreme conditions [3,4].

Military organizations comprehend an initial course for recruits, which performs a period of military adaptation, to engage soldiers’ careers [5,6]. Within this context, physical performance is necessary for conducting military operations in specific environments [7]. To maintain the highest level of physical capacity is expected that training programs could provide muscular and cardiorespiratory adaptations to prepare the soldiers for physiologic and psychological stress [8,9,10]. Effective training methods in this context include high-intensity interval training (HIIT), resistance training, and endurance training [11,12,13]. HIIT has been shown to improve both aerobic and anaerobic fitness efficiently, while resistance training is crucial for building muscle strength and endurance training enhances cardiovascular capacity [11,12]. These methods have been particularly effective in fostering the necessary adaptations in soldiers [11,12,13]. In addition, previous findings have shown that performance on military-specific tasks can prevent cardiovascular and mental diseases [14,15] and decrease musculoskeletal injuries [7,16,17] according to better long-term health [18]. Improved physical performance in the military is associated with numerous long-term health benefits, including enhanced cardiovascular health, reduced risk of metabolic syndrome, better mental health, and increased resilience to physical and mental stressors [13]. Nevertheless, it is necessary to guarantee the efficiency of the exercise program during military service. For instance, some have been shown to produce significant effects on body composition but minimal changes in total body mass [19,20], or minimal changes in physical fitness [21].

Soldiers must have excellent physical preparation, to respond to the required needs and the literature has pointed out that the profile of those who enter military service has low levels of physical fitness [22]. The level of physical performance may depend on the intensity achieved during the exercise training protocol [23,24] taking into consideration the specific tasks such as carrying bags load or war/heavy material [25]. Besides, it is also essential to observe data results according to sex differences [26]. Female soldiers generally have lower baseline fitness levels compared to male soldiers, particularly in muscular performance and body mass [26]. Research indicates that mixed-gender basic military training reduces but does not eliminate gender differences in physical fitness [27,28]. Moreover, female soldiers often face higher risks of musculoskeletal injuries compared to their male counterparts, partly due to differences in strength and body composition [27]. To evaluate the effectiveness of the implemented physical training it is essential to observe if prolonged exercise training protocols are better to promote a significant improvement in maximal oxygen uptake [29].

Given the paramount importance of physical conditioning in the military, physical training stands as a fundamental aspect of military training, including within higher education institutions such as the Instituto Superior Técnico Militar in Angola. Consequently, students at this institution undergo regular physical training throughout the academic year. The training involves significant changes in exercise routines and eating habits, potentially leading to improvements in fitness and body composition [30]. Evaluating the influence of this training on individuals’ physical fitness is essential. Despite the widespread adoption of such training, few studies have explored these changes comprehensively across both genders, prompting our research to examine outcomes between male and female cadets as well.

The current study aims to analyze the effects of a 34-week training program (equivalent to one academic year) on the body composition, muscle strength, and cardiorespiratory fitness of cadets at the Instituto Superior Técnico Militar of Angola. Furthermore, we intend to compare those effects between male and female cadets. We hypothesized that the exercise training program would result in positive changes in the variables under examination, particularly in body composition, as well as muscular and cardiorespiratory fitness. A secondary hypothesis is that male cadets will show greater improvements in muscle strength, while female cadets will show greater improvements in body composition, due to their respective higher and lower baseline fitness levels and initial body mass differences.

## 2. Materials and Methods

### 2.1. Study Design

In this pragmatic trial, participants (*n* = 74 volunteer recruits) were recruited from the Instituto Superior Técnico Militar (aged 18–26 years). These were military cadets who were engaged in their military service for 8 months, performing the usual exercise training program, comprising strength training and endurance. In the beginning, all the additional information about the research was disclosed during the recruitment process. The exercise tests have been selected for their relevance in assessing the physical condition and are therefore commonly used by conditioning sports specialists training and researchers. Each participant voluntarily provided written informed consent before participating. This study was conducted following the Declaration of Helsinki and was approved by the University of Beira Interior Ethical Board.

### 2.2. Participants

The sample consisted of 74 volunteer recruits (mean ± SD; height: 1.67 ± 0.08 m; body mass: 69.79 ± 11.01 kg; body mass index: 25.02 ± 4.04 kg/m^2^) aged between 18 and 26 years (*n* = 40 men, age: 22.13 ± 1.90 years; and *n* = 37 women, age: 20.91 ± 1.56 years), in accordance with the inclusion and exclusion criteria. Inclusion criteria to participate in the study were to be cadets at the Instituto Superior Técnico Militar of Angola, to be free of injury, and to complete all physical tests (push-ups, countermovement jumps: CMJ, sit-ups, 80-m sprint, shuttle run test, and medicine ball throwing) and anthropometric parameters (BMI, body mass, body fat, and lean mass). Subjects were asked to report any previous illnesses, injuries, or other physical problems, and were excluded if there was any evidence of an orthopedic or clinical problem or any other self-reported problem that would compromise their health or interfere with the assessment or were pregnant. Participants were carefully informed about the study design, with specific information about potential risks and discomforts that may occur.

### 2.3. Procedures

After two familiarization sessions, the evaluations were performed in six assessment sessions (48 h between each assessment) for two weeks. On the first day of testing, anthropometric measurements were determined for each participant. In the subsequent sessions, each of them performed the following physical tests: push-ups, CMJ, sit-ups, maximal oxygen uptake (VO_2_max), 80-m sprint, push-ups, and medicine ball throwing [31]. These exercises and tests have been selected for their relevance in assessing physical condition and are therefore commonly used by conditioning specialists and coaches in sports training. All study participants were familiar with the exercises.

#### 2.3.1. Body Composition

Anthropometrical and body composition data were collected at the beginning and the end of their service period during the 34 weeks of exercise training. The measurements were conducted indoors at a temperature of 22–25 °C and were consistently performed by the same researcher, a specialist in sports science. In each measure, men and women were dressed in short-sleeved underclothing. The body composition assessment was performed after waking up and the participants were instructed to avoid alcohol and vigorous exercise 24 h before evaluation.

In the first evaluation session, height was determined by a stadiometer (Seca, Hamburg, Germany), and body mass, body fat, and fat-free mass were determined by bioelectrical impedance analysis (OMRON HBF 510, Omron Healthcare, Inc., Hoffman Estates, IL, USA) [32]. Participants’ age, height, and sex were entered into the device and then the participant stepped barefoot onto it with feet width apart. Participants were instructed to hold the display unit with both hands and extend their arms parallel to the floor. Body mass index (BMI) was calculated afterward. For ethical reasons, there was no recording of menstrual cycle time in female participants.

#### 2.3.2. Push-Ups

The subject lies prone on the floor with both hands under the shoulders and pushes up off the floor until the elbows are straight while keeping the entire body straight. The subject then lowers the body with the arms until the elbows are bent at a 90° angle and the arms are parallel to the floor. Participants were instructed to repeat the exercise for 1 min and stop if they could not perform the push-up correctly [33].

#### 2.3.3. Medicine Ball Throwing

Seated on an armless chair (0.49 m) with the back straight and the medicine ball held in front of the chest, the participants had to throw the ball as far and fast as possible after instruction [31]. They performed three attempts with a 3-kg medicine ball, with a 1 min rest between each attempt. The throwing distance was measured to the nearest 0.1 cm from the chest to where the ball landed, using a flexible tape. The best result was analyzed. For the medicine ball throwing, the coefficient of variation (CV) was 4.10% and the intraclass correlation coefficient (ICC) was 0.98 (95% confidence interval, CI: 0.86–0.99) in the pre-training assessment. After training, CV was 3.95% and ICC was 0.99 (95%CI: 0.88–1.00).

#### 2.3.4. Countermovement Jump

Prior to a warm-up including some submaximal jumps, participants performed the lower body muscular strength assessment, which was assessed using the Optojump photocell system (Microgate, Bolzano, Italy). Each participant performed three CMJs with 3 min of rest between trials. All CMJs were performed with the hands on the hips throughout the test. While standing upright, participants were instructed to bend their knees to a squatting position (90°) and immediately rebound in a maximal vertical jump. No pause was allowed between the eccentric and concentric phases, and participants landed with both feet in contact with the ground. The best height score was recorded for analysis [34]. For the CMJ in the pre-training moment, the CV was 3.91%, ICC was 0.99 (95%CI: 0.90–1.00). After training, the CV was 3.30% and the ICC was 0.99 (95%CI: 0.94–1.00).

#### 2.3.5. Two Minutes Sit-Ups

Participants started with their feet and shoulders flat on the floor and their knees at a 90° angle with their arms crossed over their chest. At the beginning of the exercise, they were asked to lift their torso, bringing their chest toward their knees. When the subjects reached an angle of about 30° between their torso and the floor, they could return to the starting position. They were instructed to repeat the exercise as many times as possible for 2 min, stopping if they could not perform the sit-up correctly or if they moved their feet on the floor, it would not be counted and stopped the exercise [35].

#### 2.3.6. Curl-Ups

During the curl-up, the participants were in a standardized supine position with arms crossed over the chest, and the curl-up was performed until the shoulder blades were off the bench with a second person holding the lower legs or ankles on the floor. They were instructed to perform the exercise for 1 min, stopping if they could not perform the curl-up correctly [36].

#### 2.3.7. Sprint Test

Regarding sprint, two test trials of 80 m linear sprint running were performed (10 min of rest between trials). As a gold standard method to assess the maximal sprinting speed, 20 to 100 m running sprints are usually used [37]. The time taken to complete the 80 m distance was measured by two experienced coaches using a chronometer (Golfinho Sports MC 815, Aveiro, Portugal). The best time out of the two test trials was used for further analysis. For the sprint, the CV was 2.11%, and the ICC was 0.99 (95%CI: 0.68–1.00) in the pre-training assessment. After training, CV was 1.85% and ICC was 0.99 (95%CI: 0.70–1.00).

#### 2.3.8. Cardiorespiratory fitness

This test required running between two lines set 20 m apart at a speed dictated by a stereo system that played sounds at predetermined intervals. The initial speed was set at 8.5 km/h for the first minute and increased by 0.5 km/h each minute. The test score achieved by the subject was the number of 20-m shuttles completed before the participant either gave up voluntarily or failed to be within 3 m of the end lines on two consecutive tones [38]. VO_2_max was then calculated using a validated equation VO_2_max = (MAS × 6.65–35.8) × 0.95 + 0.182, where MAS is the maximal attained speed during shuttle run test (km/h) and VO_2_max max is the predicted maximal oxygen uptake (ml/kg/min) [39].

### 2.4. Training Program

After pretesting, each participant participated in a one-on-one explanation of the training program. All participants received a training log as well as a reference packet illustrating and describing each exercise. Exercises were explained and demonstrated to the participant and then the participant was required to perform each exercise to check for technique issues and address questions. Moreover, all sessions were supervised by drill instructors who were familiarized with the program before its commencement.

Frequency: The training program spanned 34 weeks with each week consisting of two training sessions. Participants attended these sessions as part of their mandatory physical education classes included in the curriculum of the Instituto Superior Técnico Militar of Angola.

Intensity: The intensity of exercises was individually adjustable to ensure proper training stimulus and progression. The training sessions included both low and high-intensity exercises. Specifically, endurance training involved low-intensity continuous running or marching at moderate intensity domain (55–70% of maximal heart rate), or interval training combining high (higher than 90% of maximal effort) and low-intensity running (10 to 20% of maximal effort) [40]. The calisthenics exercises were performed at maximal intended velocity and the weightlifting exercises started with 12 repetitions until failure (~70–75% of one repletion maximum load: 1RM) and progressed to 4 to 6 repetitions (~80–85% 1RM) [41]. To ensure proper training stimulus and progression, the intensity of exercises was individually adjustable, allowing participants to progressively increase the load throughout the program. Moreover, the progression (endurance and strength training component) was guaranteed each 6 to 8 weeks of training.

Time: Each training session (twice a week) lasted 90 min, starting with a standardized warm-up including low-intensity aerobic exercises, dynamic stretching, and core stability exercises for approximately 15 min [42]. The endurance and strength components were integrated within this timeframe.

Type: The training program included a mix of cardiovascular and strength components, divided into three sessions cycles. Specifically, two sessions focused more on endurance training and the next session emphasized muscular strength development. This was the structure that was followed throughout the 34 weeks of training.

As previously reported, the endurance training session included continuous running or marching at low intensity for 30 to 50 min (first to the last month of training) or interval training that combined high and low-intensity running (15 to 30 min; 1:1 work: interval ratio). After running or marching, the second part of the training session included calisthenics exercises such as jumps, push-ups, squats, pull-ups, curl-ups, and planks (circuit training, 15 to 45 s per set). The strength-oriented session started with calisthenics exercises for 5 to 15 min (e.g., jumps, push-ups, squats, pull-ups, rope climbing), followed by medicine ball throws or kettlebell swings (6 to 10 repetitions per set, 2 to 4 sets). Then, weightlifting exercises such as squats, deadlifts, clean and jerks, and bench press were performed, beginning with 12 repetitions (3 sets to muscular failure) and progressing to 4 to 6 repetitions (3 sets to muscular failure).

### 2.5. Statistical Analysis

The sample size required was computed beforehand (GPower, v.3.1.9, University of Kiel, Kiel, Germany). To detect a moderate effect size between pre and post-measures, and using an alpha of 5%, a sample size of 39 participants was needed to obtain a power of 95%. Data are presented as mean ± SD unless otherwise indicated. We used the Statistical Package for Social Sciences (IBM SPSS Statistics for Windows, version 28.0, IBM Corp., Armonk, NY, USA) to perform the statistical analysis. The Kolmogorov-Smirnov test and Levene’s test confirmed the normality and homogeneity of the data, respectively. We calculated the percentage change and 95% confidence intervals in all outcomes. The ICC (95% CI) was calculated using a two-way random effect, absolute agreement [43], while the CV was calculated as (SD/mean) × 100. Independent samples *t*-test analyzed the differences between groups at baseline and in the percentage change of outcomes. A repeated-measures ANOVA 2 × 2 (pre and post-test; male and female) with post-hoc Bonferroni tests analyzed the differences between and within groups. In addition, we performed a within-group analysis through the Student’s paired *t*-test to strengthen the analysis. We also calculated the effect size to estimate the variance between groups, using the partial eta squared (ηp^2^) and Cohen’s dz (ES) for within-subject comparisons. ES was interpreted as trivial (0.0 to 0.2), small (0.2 to 0.6), moderate (0.6 to 1.2), large (1.2 to 2.0), very large (2.0 to 4.0), extremely large > 4.0 [44]. The alpha level was set at *p* < 0.05.

## 3. Results

The changes from pre- to post-training are presented in Table 1, considering all the participants. After 34 weeks of training, significant differences were found in all assessed variables. There was a moderate decrease in body fat mass and large decreases in body mass and body mass index. On the contrary, a small increase was found in estimated VO_2_max, and moderate or large positive changes in strength-related performances, such as sprint velocity, CMJ, medicine ball throw, push-ups, and curl-ups.

At baseline, we found differences between females and males in body mass (*p* < 0.01, ES = 0.72), body fat percentage (*p* < 0.01, ES = 1.04), VO_2_max (*p* < 0.01, ES = 0.75), sprinting (*p* < 0.01, ES = 1.00), CMJ (*p* < 0.01, ES = 1.53), medicine ball throw (*p* < 0.01, ES = 0.99) and push-ups (*p* < 0.01, ES = 2.17).

**Figure 1:**
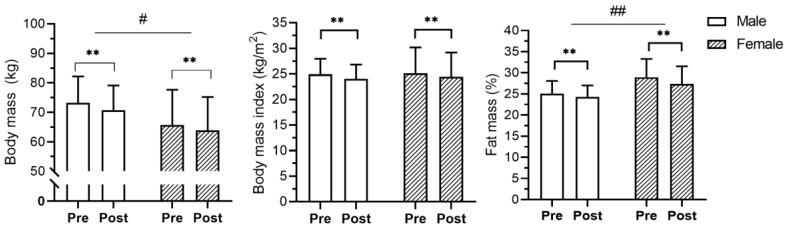
Mean and standard deviation values of anthropometrical variables before (Pre) and after (Post) the training program. ** *p* < 0.01; # *p* < 0.05 between groups; ## *p* < 0.01 between groups. Both groups reduced body mass (females 2.70% and males 3.47%), BMI (2.70% and 3.52%), and fat mass (5.34% and 3.15%).

**Figure 2:**
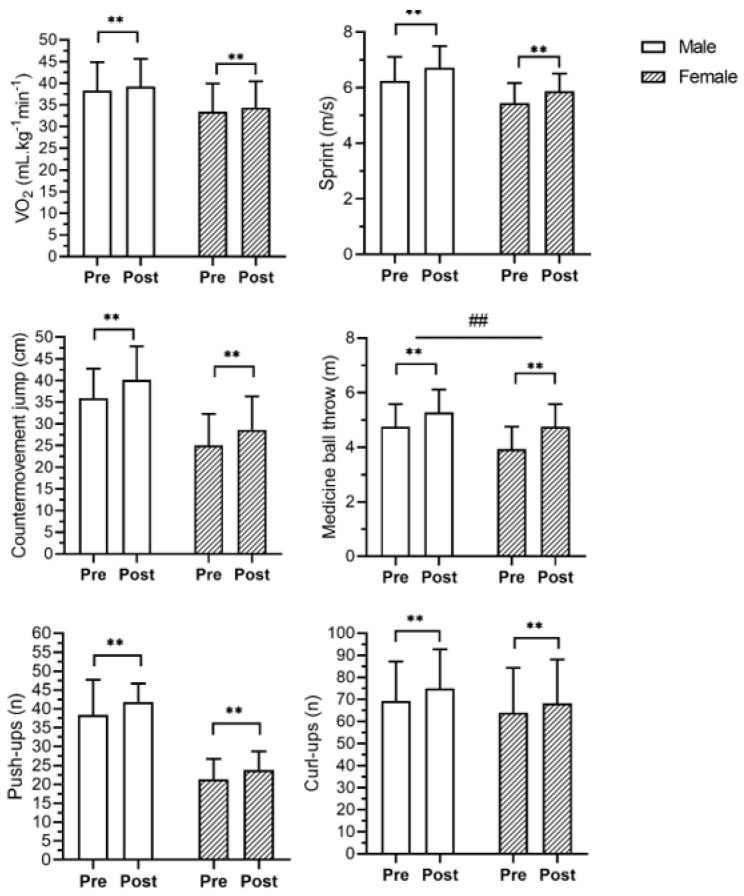
Mean and standard deviation values of physical fitness variables before (Pre) and after (Post) the training program. ** *p* < 0.01; ## *p* < 0.01 between groups. Both groups increased oxygen uptake (VO_2_max; Females: 3.28% and Males: 2.50%), sprint performance (Females: 8.46% and Males: 8.17%), countermovement jump (Females: 15.31% and Males: 11.77%), medicine ball throw (Females: 7.74% and Males: 11.47%), push-ups (Females: 14.26% and Males: 9.15%), and curl-ups (Females: 8.12% and Males: 9.14%).

Analyzing the training effects between sex, no significant interactions were found in the BMI (F = 2.40, *p* = 0.13, ηp^2^ = 0.03), VO_2_max (F = 0.02, *p* = 0.89, ηp^2^ = 0.003), sprint (F = 0.15, *p* = 0.70, ηp^2^ = 0.002), CMJ (F = 0.15, *p* = 0.70, ηp^2^ = 0.002), push-ups (F = 2.16, *p* = 0.15, ηp^2^ = 0.03) and curl-ups (F = 1.31, *p* = 0.26, ηp^2^ = 0.02). However, significant interactions were found in body mass (F = 5.18, *p* = 0.03, ηp^2^ = 0.06), body fat percentage (F = 5.31, *p* < 0.01, ηp^2^ = 0.14), and medicine ball throw (F = 10.84, *p* < 0.01, ηp^2^ = 0.13). The males presented a higher reduction of body mass while females showed a higher reduction of fat mass (Figure 1). In physical fitness variables, males showed greater improvement in medicine ball throwing than females. (Figure 1 and Figure 2).

## 4. Discussion

The current study aimed to analyze the impact of 34 weeks of typical physical training on the physical fitness of military cadets from the Instituto Superior Técnico Militar of Angola. Given the scarcity of literature addressing military training and adaptations over such an extended period [30], this research fills a crucial gap in understanding the long-term effects of military service on physical fitness. All the fitness outcomes were improved after the military service, specifically, body mass, BMI, fat mass, VO_2_max, sprint, CMJ, medicine ball throw, push-ups, and curl-ups. Among these, the greater changes were found in body mass and CMJ, medicine ball throw, and push-up performances. Interestingly, the males presented a higher reduction of body mass and greater improvement in medicine ball throwing, while the females showed a higher fat mass reduction. Overall, the 34 weeks of military service improved the physical fitness of military cadets from Angola, with a greater impact on the anthropometric and strength-related variables.

One of the most frequently studied aspects among military cadets is body composition (body mass, fat mass, and muscle mass), as it plays a pivotal role in physical performance [45]. In fact, body composition is a critical determinant of physical performance in military settings, as it directly impacts an individual’s strength, endurance, agility, and overall fitness level [21], therefore, military training programs often place a strong emphasis on achieving and maintaining optimal body composition. To achieve this goal, before designing specific military training programs, it is essential to include body composition assessments. This allows for the identification of risk factors and the profiling of individuals based on their physical condition, underscoring the importance of body composition evaluation [30].

In this study, we examined the effects of 34 weeks of physical training on body composition, revealing a reduction in fat mass. Our findings align with previous research conducted on military personnel [45,46,47]. However, we observed that overweight females experienced more significant weight loss and fat mass reduction compared to other groups. It is noteworthy that the majority of studies in this field have focused solely on young men [30,48,49], highlighting the importance of our study in providing insights into comprehensive body composition changes in military subjects of both genders. According to our results, it is evident that body composition values may vary significantly depending on the pre-training condition, indicating a robust dose-response relationship influenced by the type of exercise training undertaken [50,51]. The reductions observed in post-training body composition parameters underscore the necessity of implementing a tailored training regimen for this age group, integrating both endurance and strength tasks across all military training activities. However, based on the magnitude of our findings, we anticipate even greater reductions in fat mass.

Considering the poor level of physical activity among the evaluated Cadets of the military academy, it is important to note that the subjects under analysis are students enrolled in higher education courses affiliated with the military academy, including medicine, electrical engineering, civil engineering, mechanical engineering, among others. They are not regular soldiers, and their roles after graduation cannot be directly compared to those of regular soldiers, as they will become specialized officers. Furthermore, as university students who spend a significant amount of time studying (a typically sedentary activity), they may not prioritize the development of their physical abilities to the same extent. This context may help justify the low level of physical activity observed in the population.

Another factor contributing to the initial low fitness performance is the significant role of military service as a crucial aspect of security for individuals and their families in certain countries like Angola, providing an avenue for life transformation [52]. Consequently, individuals from various backgrounds, including those with lower physical conditioning, aspire to enroll in military institute. Although exercise training protocols spanning 8 to 12 weeks of intervention have demonstrated comparable outcomes [30], given the duration of our protocol, it was anticipated that more significant improvements would be attained.

The reduction in cardiorespiratory fitness showed a similar pattern. The extent of improvement observed aligns with findings from previous research [53,54], but we expected a more significant improvement. This outcome may be influenced by the testing method employed, which involves sprints with directional changes. Higher running intensities entail greater acceleration changes, including stopping, changing direction, and restarting, which can lead to increased fatigue [55]. Similarly, future studies should incorporate aerobic training into the exercise regimen, particularly focusing on variables related to aerobic fitness [55]. Interestingly, moderate enhancements were noted in both female and male cadets during the sprint test, suggesting that daily physical activities, such as heavy weightlifting in the lower limbs (e.g., carrying bags or running), could contribute to these improvements outside the formal exercise training program, potentially affecting cardiorespiratory outcomes. Once again, it is plausible that the exercise regimen implemented may not have been sufficient to induce such changes.

In terms of muscular performance, it is noteworthy that all physical fitness variables did not exhibit gender differences, except for the medicine ball throwing test, which demonstrated greater improvement in males compared to females. This variance could stem from several factors, including potentially higher motivation or stimulation among male cadets during the exercise test, or possibly, a greater adaptation to strength training specifically targeting the upper limbs. It is important to acknowledge that while some studies may share similar training variables with our research, there may be differences in the duration dedicated to endurance and strength training [30], making comparisons challenging across different exercise training protocols. Our study spanned 34 weeks of combined training, which led to positive adjustments in the performance of military cadets and surpassed the duration of most research in this field. This longer intervention period may have contributed to the observed weight loss compared to previous studies. Conversely, since our results should ideally surpass those of recent studies with shorter intervention periods, this may further elucidate the significant improvements in upper-limb muscular strength observed in the medicine ball throwing and CMJ tests.

Based on our findings, medicine ball throwing and CMJ tests appear relevant for assessing upper and lower body strength and power in young cadets compared to traditional tests like push-ups and curl-ups. This discrepancy could be attributed to these tests being less familiar to military recruits, potentially leading to technical improvements acquired between pre and post-training assessments [21].

Additionally, it is imperative to comprehend the relationship between absolute strength, power, and muscular endurance. While the strength tests employed varied in duration and muscle groups engaged, they were nevertheless swift and straightforward to administer [30]. Both the medicine ball throwing and CMJ exercises exhibit several characteristics, including the recruitment of fast-twitch muscle fibers [21], which may result in increased muscle force production. This enhanced capacity can be directly applicable to essential military activities during combat or training scenarios, such as rapid movements, reactive responses, and swift manipulation of equipment.

Based on the preceding discussion, all facets of physical fitness components displayed improvement following the training regimen, yet certain variables within the program, such as load, intensity, duration, repetitions, and participant age, may impact the outcomes [30]. Furthermore, when comparing female and male cadets aged between 18 and 26 years, variations in anthropometric characteristics can affect the analysis of body composition. The findings of this study offer initial evidence that typical physical training significantly contributes to reducing fat mass and enhancing muscular performance, taking into account the pre-training attributes of both male and female cadets in Angola. However, it appears that comprehensive characterization at the outset and the utilization of tailored assessment tests are essential for devising a well-tailored training protocol that addresses gender-specific adjustments for military cadets. Gender-specific interventions could prove beneficial in refining these attributes to optimize physical performance and mitigate intersex variability in muscular or aerobic components, which is crucial in military occupational roles [56]. Investigating the impact of a typical, established exercise training program on fitness capacity may assist the military in enhancing recruits’ performance and readiness for combat or other military scenarios.

Given the initial low fitness levels, particularly among female cadets, there is a critical need for pre-training conditioning that prepares recruits for the rigors of such intensive programs. We assessed the impact of the typical military training program conducted over an academic year at the Instituto Superior Técnico Militar of Angola. It was concluded that the expected improvements in cardiorespiratory capacity and muscular strength were not achieved. This outcome prompts a reflection on the need to modify the training regimen for these Cadets, both in terms of training volume and the type of exercises employed. This study sets the groundwork for developing guidelines for structuring military training for Cadets in the Angolan army. This research supports the need for ongoing adaptation and innovation in military training practices to ensure they remain effective and relevant. Continued research into different training intensities, durations, and gender-specific adaptations is recommended to further refine these training programs.

This study has several limitations. It is crucial to control hydration and dietary intake before assessing body composition, especially considering the menstrual cycle for female participants. Additionally, the inclusion of a control group would facilitate comparisons to evaluate the effectiveness of the exercise training protocol. Furthermore, BMI alone is an inadequate indicator of body fat and should be supplemented with comprehensive evaluations that encompass all body compartments, particularly fat or fat-free mass. Future research should design tailored exercise training programs aimed at improving endurance and strength performance, while considering the pre-training characteristics of military personnel. Moreover, the study advocates for further research in this area, particularly investigating various exercise training intensities and durations, as well as exploring the psychological and physiological benefits of military-style training for healthcare personnel and fitness performance.

## 5. Conclusions

The current study demonstrated that structured military training, encompassing both strength and endurance components, significantly enhances physical fitness among military cadets, improving their readiness for the demands of military service. The findings affirm that comprehensive training programs, which are responsive to the physiological differences between genders, can effectively increase muscle mass, reduce fat mass, and enhance overall physical performance. However, larger improvements in cardiorespiratory capacity and muscular strength were expected. This highlights the need for the development of new training guidelines for students (e.g., from the Instituto Superior Técnico Militar of Angola), with a focus on ensuring safety and enhancing fitness performance among cadets of both genders.

## Figures and Tables

**Table 1:** Changes in variables from pre- to post-test in anthropometrics, cardiorespiratory, and muscular fitness variables (*n* = 74).

	Pre-Training	Post-Training	Δ (95% CI)	*p*-Value	ES
Body mass (m)	69.79 ± 11.01	67.56 ± 10.35	2.23 (0.35)	<0.01 *	1.48 [large]
Body mass index (kg/m^2^)	25.02 ± 4.04	24.21 ± 3.80	0.81 (0.12)	<0.01 *	1.48 [large]
Fat mass (%)	26.86 ± 4.13	25.71 ± 3.78	1.16 (0.23)	<0.01 *	1.14 [moderate]
VO_2_max (mL/kg/min)	36.09 ± 6.90	36.99 ± 6.67	0.90 (0.43)	<0.01 *	0.48 [small]
Sprint (m/s)	5.88 ± 0.89	6.33 ± 0.83	0.45 (0.10)	<0.01 *	1.08 [moderate]
CMJ (cm)	30.92 ± 8.87	34.86 ± 9.60	3.94 (0.39)	<0.01 *	2.33 [large]
Medicine ball throw (m)	4.38 ± 0.92	4.80 ± 0.99	0.42 (0.07)	<0.01 *	1.31 [large]
Push-ups (*n*)	30.51 ± 11.56	33.57 ± 12.21	3.05 (0.59)	<0.01 *	1.21 [large]
Curl-ups (*n*)	66.88 ± 18.99	71.92 ± 18.82	5.04 (1.30)	<0.01 *	0.90 [moderate]

* *p* < 0.01.

## Data Availability

The raw data supporting the conclusions of this article will be made available by the authors on request.
